# Actual exclusive breastfeeding rates and determinants among a cohort of children living in Gampaha district Sri Lanka: A prospective observational study

**DOI:** 10.1186/1746-4358-7-21

**Published:** 2012-12-22

**Authors:** Priyantha J Perera, Nayomi Ranathunga, Meranthi P Fernando, Wikum Sampath, Gayni B Samaranayake

**Affiliations:** 1 Department of Paediatrics, Faculty of Medicine, Ragama, Sri Lanka

## Abstract

**Background:**

Exclusive breastfeeding (EBF) during the early months of life reduce infant morbidity and mortality. Current recommendation in Sri Lanka is to continue exclusive breastfeeding up to six months of age. Exclusive breastfeeding rates are generally assessed by the 24 recall method which overestimates the actual rates. The objective of this study was to determine actual exclusive breast feeding rates in a cohort of Sri Lankan children and to determine the reasons that lead to cessation of breastfeeding before six months of age.

**Methods:**

From a cohort of 2215 babies born in Gampaha district, 500 were randomly selected and invited for the study. They were followed up at two (n = 404), four (n = 395) and six (n = 286) months. An interviewer administered questionnaire asked about feeding history and socio-demographic characteristics. Child health development record was used to assess the growth.

**Results:**

Exclusive breastfeeding rates at two, four and six months were 98.0%, 75.4% and 71.3% respectively. The main reasons to stop exclusive breastfeeding between two to four months was concerns regarding weight gain and between four to six months were mothers starting to work. Majority of the babies that were not exclusively breastfed still continued to have breast milk. Mothers above 30 years had lower exclusive breastfeeding rates compared to younger mothers. Second born babies had higher rates than first borns. There was no significant association between maternal education and exclusive breastfeeding rates.

**Conclusions:**

Exclusive breastfeeding rates were high among this cohort of children. A decrease in EBF was noted between two and four months. EBF up to six months does not cause growth failure. Mothers starting to work and concerns regarding adequacy of breast milk were the major reasons to cease EBF. The actual exclusive breastfeeding rates up to six months was 65.9%.

## Background

Exclusive breastfeeding during early months of life reduces infant morbidity and mortality [1]. World Health Organization (WHO) defines exclusive breastfeeding as, ‘feeding an infant only with breast milk, excluding solids or any other fluids (including infant formulas) except medicines, vitamins, and minerals’ [2]. In 2001, WHO recommended infants to be exclusively breastfed during first 6 months of life [3]. The main issue pertaining exclusive breastfeeding is how long it supports nutrition of a rapidly growing infant.

The WHO recommendation is in effect in Sri Lanka since 2005. Before this the recommendation was to continue exclusive breastfeeding up to 4–6 months. According to Sri Lanka Demographic & Health Survey (DHS) 2006/07, the exclusive breastfeeding rate in Sri Lanka between 0 – 5 months was 75.8% [4]. The 24 hour recall method was used in this survey, which is the standard method of assessing exclusive breastfeeding rate. According to this method even a child who had other foods, but only breastfed within 24 hours of inquiry, is considered exclusively breastfed. Agampodi et al. have shown that actual exclusive breastfeeding rates are lower than those derived from the 24 hour recall method [5]. Another Sri Lankan study by Perera et al. assessed feeding practices of children between 2 to 5 years in retrospect and found 62% of babies were exclusively breastfed up to 6 months [6]. Limitation of retrospective studies, compared to 24 hour recall method is the higher recall bias. The most accurate way to calculate actual exclusive breastfeeding rate is to follow up a cohort of babies periodically up to 6 months of age. A high dropout rate is the main limitation in this type of a study.

Sri Lanka is a low middle income country, with impressive health statistics. Maternal mortality ratio (33.4 per 100,000) and infant mortality rate (10.1 per 1000 live births) are among lowest for the region [7]. According to Sri Lanka labour force survey 2010, 31.1% of females living in Gampaha district were economically active. This includes both formal and informal employment. Sri Lankan mothers employed in government sector are entitled to full pay maternity leave of 84 working days, excluding weekends and public holidays. This is equivalent to about 4 calendar months in Sri Lanka. If a mother wishes to extend maternity leave, she has to go on half pay, followed by no pay leave. Lactating mothers working in private sector do not receive the same privileges.

The main objective of this study was to assess actual rates of exclusive breastfeeding at different ages and to find out reasons for ceasing exclusive breastfeeding before six months of age. Exclusive breastfeeding rates were satisfactory among the cohort of children included in the study. Further improvement in exclusive breastfeeding rates seems to be possible with simple interventions.

## Methods

Sri Lanka consists of 25 administrative districts. Gampaha district is the 2nd highest populated district after Colombo, with about 12% of the country’s population. Gampaha is only second to Colombo district in socioeconomic standards. In 2007, 28,848 babies were born in Gampaha district [8]. Gampaha consists of a socio-economically mixed population.

### Participant selection

Previously, a study was conducted by authors of this paper, between October and November 2010 to calculate statistical characteristics of growth parameters at birth of babies born in Gampaha district. Almost all babies born at full term in Gampaha district during the study period (n = 2215 babies) were included. A full term baby was defined as “a baby born after 37 weeks of gestation, without any conditions affecting antenatal growth”. A follow up study was conducted with a random sample of the 500 babies. Computer generated numbers were used to select the sample. Selected children were assessed periodically regarding breastfeeding practices and growth. Exclusive breastfeeding rate in Sri Lanka was around 70% according to DHS survey. A sample size of 281 was required to estimate this prevalence within 5% (prevalence 70% and 95% confidence interval 65% to 75%). Assuming 80% of the babies will join the study initially and about 80% will complete the study, 500 babies were selected for the study.

### Data collection

Parents were contacted either over the phone or through mail informing the place, time and date of follow ups. For the follow ups a special clinic was conducted twice a week, at the University Paediatric Unit of Teaching Hospital, Ragama. Babies were followed up at two, four and six months of age, irrespective of their feeding practices. At each follow up feeding practices of the baby during previous two months were recorded using a pretested, validated questionnaire administered by an interviewer. Mothers were specifically questioned whether any food, formula milk or water was given to the baby, other than breast milk during the period concerned. At each follow up, babies who were only on breast milk up to that time were considered exclusively breastfed up to that age. A baby who was given any food, drink or water, in addition to breast milk was considered not exclusively breastfed. Medicines, vitamins, minerals and oral rehydration solution (ORS) given on medical advice were allowed. Socio-demographic data were obtained from data collected for the original study.

When baby was not exclusively breastfed, the reasons to stop exclusive breast feeding were obtained from the mother and recorded. If a mother mentioned inadequate weight gain as the cause of growth faltering, the child health development record of the child was checked to verify whether growth faltering was present. When there was no clear growth faltering, that was recorded as mother felt inadequate breast milk category. At each clinic advantages of breastfeeding were explained to mothers by the consultant/senior registrar. Mothers were encouraged to restart exclusive breastfeeding when there was no clear reason to stop exclusive breastfeeding. Working mothers were educated about expressing breast milk.

Father’s occupation was categorized according to the International Standard Classification of Occupation (ISCO-08) by international labour organization [9]. According to this, legislators and different types of managers are category 1, labourers are category 9 and armed services are category 0. Highest education achieved by parents was recorded. In Sri Lanka, students achieve General Certificate of Education, Ordinary Level (equivalent to GCSE in the UK), at grade 11. Maternal age was age of the mother in completed years at the time of delivery.

All children were examined by a consultant Paediatrician or a senior registrar in Paediatrics. Children with health concerns were managed accordingly. Parents did not have extra expenses other than for travelling. Mothers were informed the date of next follow up. Reminders were also sent via phone calls or postcards.

### Data analysis

The Statistical Package for Social Sciences (SPSS) version 16 was used to analyse data. Exclusive breastfeeding rates between different groups were compared with either ANOVA or t tests. Significance was set at p < 0.05 (Significance level 95%).

### Ethical issues

At the time of recruitment for the original study, all mothers were informed about the follow up study. Informed written consent was re-obtained from mothers at the first follow up, after explaining the objectives of the study. Participation was entirely voluntary and mothers had the liberty of withdrawing from the study at any time. Advantages of exclusive breastfeeding were emphasized and incorrect feeding practices were corrected. Ethical approval for the study was obtained from the ethics committee of Faculty of Medicine, University of Kelaniya (P93/09/2010).

## Results

Of the 500 babies invited, 404, 395 and 286 babies were present at two, four and six months respectively. Babies who completed all three follow ups had mixed socio-demographic characteristics as summarised in Table 1. 

**Table 1:** Socio-demographic characteristics of the study population

**Characteristic**	**Frequency**
**Father’s occupation**	
Category 1	10 (3.5%)
Category 2	07 (2.4%)
Category 3	11 (3.8%)
Category 4	18 (6.3%)
Category 5	59 (21.0%)
Category 6	12 (4.1%)
Category 7	62 (21.5%)
Category 8	60 (21.0%)
Category 9	37 (12.9%)
Category 0	10 (3.5%)
**Mother’s employment status**	
Formally employed	49 (17.3%)
Not formally employed	237 (82.7%)
**Mother’s age**	
**<**30 years	152 (53.1%)
>30 years	134 (47.9%)
**Father’s education**	
Up to grade 5	07 (2.5%)
Grade 6 to 11	191 (67.1%)
Grade 12 to 14	79 (27.3%)
University	09 (3.1%)
**Mother’s education**	
None	01 (0.4%)
Grade 1 to 5	06 (2.1%)
Grade 6 to 11	180 (62.9%)
Grade 12 to 14	90 (31.5%)
University	09 (3.1%)
**Monthly family income(SLR)**	
< 9,999	19 (6.6%)
10,000 - 19,999	104 (36.4%)
20,000 - 34,999	97 (33.9%)
>35,000	66 (23.1%)

Within the first two months of life 98% of babies were exclusively breastfed. Exclusive breastfeeding rates among babies presenting at two, four and six months of age are illustrated in Table 2. All babies who were not exclusively breastfeeding at two months presented for the follow up at four months. At four months, 97 babies of 395 were not on exclusively breastfeeding. Of this, 89 babies stopped exclusive breastfeeding between two and four months, giving a dropout rate of 23% in exclusive breastfeeding during this period. At six months, of the 286 babies presented for follow up 82 were not being exclusively breastfed. Of these 82 babies, 30 stopped exclusive breastfeeding between four and six months, giving a dropout rate of 12.8% for this period. Accordingly, total dropout rates of exclusive breastfeeding between birth and six months is 34.1%, which results in the actual exclusive breastfeeding rate of 65.9%. 

**Table 2:** Exclusive breastfeeding rates at different ages

**Age of babies**	**Exclusively breastfed**	**Exclusive breastfeeding rate**
2 months (n = 404)	396	98.0%
4 months (n = 395)	298	75.4%
6 months (n = 286)	204	71.3%

Of the babies not exclusively breastfed, the majority continued breastfeeding along with formula milk or complementary feeds. At four months, most babies not exclusively breastfeeding were having breast milk plus infant formula, and at six months they were having breast milk and complementary feeds. The feeding pattern of babies not exclusively breastfed is summarised in Table 3. 

**Table 3:** Pattern of feeding among babies who were not exclusively breastfed at 2, 4, and 6 months of age

**Age**	**Stopped breastfeeding totally**	**Breastfeeding + infant formula**	**Breastfeeding + complementary feeds**	**Breastfeeding + other feeds**
2 months (n = 08)	01 (12.5%)	07 (87.5%)	0	0
4 months (n = 97)	17 (17.5%)	72 (74.2%)	07 (7.2%)	01 (1.1%)
6 months (n = 82)	15 (18.3%)	27 (32.9%)	33 (40.2%)	07 (8.5%)

Between birth and two months, maternal anxiety of inadequate breast milk was the main reason to stop exclusive breastfeeding, while inadequate weight gain was the commonest reason between two and four months. Mother starting to work was the commonest reason between four and six months. Main reasons to stop exclusive breastfeeding at different ages are summarised in Table 4. 

**Table 4:** Reasons to abandon exclusive breastfeeding at 2, 4 and 6 months of age

**Age period**	**Mother felt not enough breast milk**	**Inadequate weight (growth faltering)**	**Mother returning to work**	**Other conditions in mother or baby**
0-2 month (n = 08)	04 (50%)	02 (25.0%)	00 (0.0%)	02 (25%)
2-4 months (n = 89)	22 (24.7%)	45 (50.6%)	19 (21.3%)	03 (3.4%)
4-6 months (n = 30)	00 (0.0%)	07 (23.3%)	23 (76.7%)	00 (0.0)

Second born babies had a higher exclusive breastfeeding rate compared to first borns. The rate dropped again after the second baby. No statistically significant association was found between exclusive breastfeeding and birth order. The association between birth order and exclusive breastfeeding is depicted in Table 5. 

**Table 5:** Relationship between birth order and exclusive breastfeeding up to 6 months

**Birth order**	**Number exclusively breastfed up to six months**	**Exclusive breastfeeding rate**
1^st^ baby (n = 120)	84	70.0%
2^nd^ baby (n = 106)	78	73.6%
After 2^nd^ baby (n = 60)	40	66.6%

Of 286 mothers, 152 (53%) were less than 30 years of age. The exclusive breastfeeding rate in these women was 76.9%, while in mothers over 30 years of age it was 63.2%. The difference in exclusive breastfeeding rate with maternal age was statistically significant (p < 0.01). Mothers studied only up to GCE (O/L) had a higher exclusive breastfeeding rate (80%) compared to mothers who had a higher education (68.6%), but the difference was not statistically significant.

## Discussion

According to this study exclusive breastfeeding rates at six months in a cohort of 286 Sri Lankan children living in a mixed urban and suburban area was 71.3%. However, on longitudinal analysis only 65.9% of babies were exclusively breastfed up to six months. The discrepancy resulted from disproportionately higher number of babies not on exclusive breastfeeding at four months, failing to attend the final follow up. Of the babies presented at four months, 72.4% were presented at six months, but of the babies not exclusively breastfed at four months, only 53.6% were presented at six months. This highlights the inaccuracy of estimating exclusive breastfeeding rate by cross sectional studies.

According to the UNICEF, only every third child living in the developing world is exclusively breastfed during first six months of life [10]. According to our study and previous studies [4,6], exclusive breastfeeding rates in Sri Lanka are much higher than quoted by UNICEF. The high exclusive breastfeeding rates recorded is probably due to dedication of health care workers and the commitment of government towards promoting exclusive breastfeeding. High female literacy rate in Sri Lanka is another important reason [11]. A breastfeeding code promoting exclusive breastfeeding is in effect in Sri Lanka. A committee of experts and officials monitor activities related to infant feeding for any violations of the code. Appropriate action is taken against offenders.

Even under optimum conditions it is not possible to achieve 100% of exclusive breastfeeding due to other factors involved [10]. Postpartum complications or medication taken by the mother may prevent breastfeeding. Occasionally there are some mothers who may need additional support to feed their babies. Failure in breastfeeding is due to incorrect feeding techniques compared to inadequacy of breast milk in most instances. Maternal anxiety due to fear of inadequate breast milk is an important factor for failure of lactation. Reassuring the mother and correcting the technique is sufficient in most cases of lactation failures. Lactation management centres established in some hospitals in Sri Lanka, play a major role in promoting exclusive breastfeeding. Fifty percent of the babies not exclusive breastfed at two months and 24.7% of the babies not exclusive breastfed at four months were started on formula milk or complementary food based on the mothers own judgment. This tendency was reported in a previous study as well [6].

When exclusive breastfeeding up to six months was implemented, the main concern was its ability to support growth up to six months. In our study a higher number of mothers had stopped exclusive breastfeeding due to growth faltering between two and four months than at four and six months. Growth charts used in Sri Lanka are based on WHO Multicentre Growth Reference Study (MGRS). The theory behind MGRS was that ‘growth of children from birth to five years depends mainly on nutrition, feeding practices, environment and health care than genetics or ethnicity [12]. Some studies have shown that genetic factors do play a role in determining growth of a child [13]. Two studies from Sri Lanka, recorded the mean birth weight of Sri Lankan children to be 2.8 and 2.9 kg respectively [14,15]. This falls on the minus one standard deviation of MGRS charts. Therefore, we can assume growth charts based on MGRS are not appropriate for growth monitoring in Sri Lanka. Due to wrong interpretation of growth, some Sri Lankan children may be losing benefits of exclusive breastfeeding. We suggest that the decision to stop exclusive breastfeeding due to growth faltering should be made by a doctor with careful consideration of possible genetic influences.

A significant decrease in exclusive breastfeeding between four and six months was due to mothers commencing work. Expressed breast milk is an alternative only for few mothers as numbers of hours spent out of home is too long. In Sri Lanka, full pay maternity leave for lactating government workers cover only four calendar months and mothers who wish to extend maternity leave need to go on half or no pay leave. In the private sector maternity leave is less than this, because private sector employers have their own regulations regarding maternity leave. There are significant numbers of mothers engaged in informal employment, who receive no maternity benefits. If exclusive breastfeeding rates are to be improved maternity leave needs to be extended up to six months. An allowance paid to mothers not formally employed, but willing to continue exclusive breastfeeding until six months will also have a positive impact on exclusive breastfeeding. However, the main obstacle in implementing such changes are the economical constrains.

According to data from western countries older mothers have higher breastfeeding rates [16,17]. In our study, mothers over 30 years of age had a lower exclusive breastfeeding rate compared to mothers less than 30 years of age. Higher employment rates among older mothers and increased work load at home may be possible reasons. Definite explanation cannot be given as we have not studied reasons for the lower exclusive breastfeeding rates among mothers over 30 years of age.

Mothers educated above GCE (O/L) had a lower exclusive breastfeeding rate compared to mothers who had a lower education levels. Although mothers with higher education are more likely to know benefits of exclusive breastfeeding, they are more likely to be employed as well. In our study, 21 out of 26 mothers who stopped exclusive breastfeeding based on their own judgement were educated above grade 11. Educated mothers seem to be more anxious about adequacy of breast milk, resulting in a lower threshold to stop exclusive breastfeeding.

First time mothers are more likely to have difficulties in establishing breastfeeding and often more anxious due to lack of experience. With the second born, they will be more confident about breastfeeding. Increased maternal age, increased house hold work, presence of other children and higher employment rates, may be contributing for the lower rate of exclusive breastfeeding after the second child.

According to the DHS done in 2006/07, exclusive breastfeeding rate in Sri Lanka between 0 to 5 months was 76% [4]. A study done in 2009, in an area belonging to Colombo district, Sri Lanka, revealed an exclusive breastfeeding rate of 77.5% among babies between 4 to six months [18]. In both these studies babies were recruited without selection and 24 hour recall method was used to assess exclusive breastfeeding rates. Children included in our study were born at term, without any medical problems. The main reason for the lower exclusive breastfeeding rates in our study is due to babies up to six months were included compared to other two studies. The populations involved in previous studies and time frame were also different from our study.

This study shows that assessing exclusive breastfeeding rates through a cross sectional study is subjected to errors. Even when a sample of children are followed up prospectively the exclusive breastfeeding rates may differ from actual figures due to drop outs. This is highlighted by this study. Exclusive breastfeeding rate among babies followed up till six months was 71.3% due to disproportionately higher number of babies not on exclusive breastfeeding dropping out from the study between four and six months. Therefore, this rate was exaggerated. On longitudinal analysis, dropout rate in exclusive breastfeeding was 34.1%, giving 65.9% of actual exclusive breastfeeding rate up to six months.

### Limitations

The major limitation of this study was high dropout rates. All attempts were made to minimize the dropout rates. Reminders were given about the follow up. Defaulted mothers were contacted and an alternate date was given. We did a longitudinal analysis on dropout rates to overcome the error due to dropouts. We compared the socio-demographic characteristics of dropouts with that of children who completed the follow up and found there was no significant difference.

## Conclusions

The exclusive breastfeeding rates are satisfactory in this cohort of children compared to figures from other developing countries. The greatest decrease in exclusive breastfeeding was noted between two and four months of age. Main reasons to abandon exclusive breastfeeding were mothers commencing work, maternal anxiety about adequacy of breast milk and growth failure. Younger mothers are more likely to practice exclusive breastfeeding than older mothers. Maternal education does not seem to have a significant influence on exclusive breastfeeding rates. Actual exclusive breastfeeding rate in this cohort of children was 65.9%. Further improvement in exclusive breastfeeding is possible in Sri Lanka with simple interventions.

## Competing interests

All authors declare there are no competing interests. No funding was obtained to conduct the study from any agency.

## Authors’ contributions

PJP was involved in planning, supervising data collection, data analysis and preparation of the manuscript. MPF and NR were involved in planning, data collection and preparation of the manuscript. GBS and WS were involved in planning, data analysis and preparation of the manuscript. All authors have gone through the final version of manuscript and approved submission for publication.
